# Maternal vitamin B12, vitamin D, and folic acid status during pregnancy and child neurodevelopment: a systematic review

**DOI:** 10.3389/fnins.2026.1825297

**Published:** 2026-06-10

**Authors:** Lívia Franco, Camila Vilhena Furquim de Andrade, Laís Virgílio Margini, Nuno Sousa

**Affiliations:** 1 Centro Universitário Max Planck (UniMAX), Indaiatuba, SP, Brazil; 2 Centro Universitário de Jaguaríuna (UniFAJ), Jaguaríuna, SP, Brazil

**Keywords:** child neurodevelopment, cognition, folic acid, maternal nutrition, vitamin B12, vitamin D

## Abstract

**Background:**

Adequate maternal micronutrient status during pregnancy may play an important role in fetal neurodevelopment. Emerging evidence suggests that deficiencies in vitamin B12, vitamin D, and folic acid, often exacerbated by modern dietary patterns characterized by high consumption of ultra-processed foods, may adversely affect cognitive, motor, and behavioral outcomes in offspring. Despite growing interest in this topic, the magnitude and consistency of these associations remain incompletely defined.

**Methods:**

We conducted a systematic review in accordance with PRISMA 2020 guidelines. Electronic databases (PubMed, Cochrane Library, LILACS, and ScienceDirect) were searched for studies published up to March 2025 that evaluated maternal vitamin B12, vitamin D, or folate status during pregnancy and subsequent neurodevelopmental outcomes in children. Eligible studies included observational studies and randomized controlled trials assessing cognitive, motor, and behavioral domains.

**Results:**

A total of 22 studies met the inclusion criteria, comprising 18 observational studies (81.8%) and 4 randomized controlled trials (18.2%). Vitamin D was the most frequently investigated micronutrient, followed by vitamin B12 and folate-related exposures. Adequate maternal vitamin B12 status was consistently associated with improved language, memory, and cognitive performance, while vitamin D deficiency during pregnancy was linked to poorer motor and neurocognitive outcomes. Folic acid supplementation, particularly when initiated in the periconceptional period, demonstrated the most consistent favorable associations extending beyond neural tube defect prevention, including improved cognitive and language development. However, substantial methodological heterogeneity limited quantitative synthesis.

**Conclusion:**

Maternal vitamin B12, vitamin D, and folate status appear to play an important role in shaping early neurodevelopment. In the context of increasing consumption of ultra-processed foods and declining micronutrient density in modern diets, these findings underscore the need for strengthened nutritional surveillance, targeted supplementation strategies, and updated public health policies to support optimal neurodevelopmental outcomes.

## Introduction

Maternal nutrition is a central determinant of fetal brain development, influencing neurogenesis, synaptogenesis, myelination, and long-term cognitive function. During pregnancy, rapid cellular proliferation and neural differentiation create heightened vulnerability to nutritional inadequacies. Deficiencies in key micronutrients - particularly vitamin B12, vitamin D, and folic acid - have been increasingly implicated in adverse neurodevelopmental trajectories, with potential long-term consequences for cognition, behavior, and neurodevelopment (Prado and Dewey, 2014; Cusick and Georgieff, 2016; Nyaradi et al., 2013).

Vitamin B12 plays a critical role in one-carbon metabolism, DNA synthesis, and myelin formation. Insufficient maternal B12 levels have been associated with impaired neuronal maturation, altered neurotransmitter synthesis, and disruptions in cognitive and language development. Evidence from cohort studies indicates that children born to mothers with suboptimal B12 status exhibit lower cognitive performance, reduced attention, and poorer academic outcomes (Black, 2008; Dror and Allen, 2008; Veena et al., 2010; Aparicio et al., 2020).

Folate plays an essential role in neural tube closure and epigenetic regulation during early embryogenesis. Beyond its established role in preventing neural tube defects, accumulating evidence indicates that folate availability influences neurogenesis, synaptic plasticity, and long-term cognitive function. Periconceptional folic acid supplementation has been associated with improved language development, executive functioning, and reduced risk of neurodevelopmental disorders (Czeizel et al., 2011; Julvez et al., 2009; Roth et al., 2011; Surén et al., 2013).

Vitamin D, traditionally linked to bone health, is now recognized as a neuroactive steroid involved in neurogenesis, neuroprotection, and modulation of neurotransmission. Vitamin D receptors and metabolizing enzymes are widely expressed in the developing brain. Maternal vitamin D deficiency has been associated with increased risk of adverse neurodevelopmental outcomes, including impaired motor development, reduced cognitive performance, and increased risk of behavioral disorders (McCann and Ames, 2008; McGrath et al., 2010; Morales et al., 2015; Strøm et al., 2014).

Importantly, these micronutrient deficiencies occur within a broader nutritional transition characterized by increased consumption of ultra-processed foods. Such diets are typically energy-dense yet poor in essential micronutrients, contributing to “hidden hunger” even in high-income settings. This nutritional shift may exacerbate maternal micronutrient inadequacy during critical windows of fetal development, amplifying risks to neurodevelopment (Bailey et al., 2015; Black et al., 2013).

Against this background, the present systematic review aims to synthesize current evidence on the relationship between maternal vitamin B12, vitamin D, and folate status and child neurodevelopmental outcomes. By integrating findings across observational and interventional studies, this review seeks to clarify biological mechanisms, identify gaps in the literature, and inform nutritional strategies and public health policies aimed at optimizing maternal and child health.

## Methods

### Inclusion and exclusion criteria

This systematic review included randomized controlled trials (RCTs) and observational studies (prospective or retrospective cohort and cross-sectional designs) that examined the association between maternal vitamin B12, vitamin D, and/or folate status (biomarkers and/or supplementation) during pregnancy and offspring neurodevelopmental outcomes assessed from birth to 5 years of age. Eligible studies were required to (i) include pregnant participants, (ii) measure maternal exposure during pregnancy (serum/plasma biomarkers and/or supplementation), and (iii) report at least one child neurodevelopmental outcome assessed using standardized or clearly described instruments.

To enhance comparability across studies, vitamin “deficiency” was interpreted using internationally accepted biochemical thresholds whenever possible: vitamin B12 < 200 pmol/L, 25(OH)D < 50 nmol/L (20 ng/mL), and folate <7 nmol/L (serum) or <305 nmol/L (RBC folate). When studies used alternative cut-offs, the definitions reported by the authors were retained and documented for interpretation (EFSA Panel on Dietetic Products, Nutrition and Allergies (NDA), 2014; EFSA Panel on Dietetic Products, Nutrition and Allergies (NDA), 2015; EFSA Panel on Dietetic Products, Nutrition and Allergies (NDA), 2016).

Neurodevelopmental outcomes of interest were prespecified and grouped into the following domains:

- Cognitive development: global cognition, IQ, executive function, attention, memory, processing speed.
- Language development: receptive and expressive language, communication milestones.
- Motor development: gross and fine motor skills, developmental milestones.
- Socio-emotional/behavioral outcomes: behavioral problems, emotional regulation, internalizing/externalizing symptoms, and clinically defined neurodevelopmental or behavioral disorders when reported (e.g., ADHD- or ASD-related outcomes).

Studies were excluded if they: (i) were conducted in animals; (ii) were not peer-reviewed (e.g., conference abstracts without full text); (iii) did not provide quantitative exposure and outcome data; (iv) focused exclusively on populations outside the scope (e.g., non-pregnant adults, older adults); or (v) addressed conditions unrelated to the review objective (e.g., studies where neurodevelopmental outcomes were not reported or could not be extracted). Systematic reviews and meta-analyses were screened to support contextual discussion but were not included in the primary study count unless they provided extractable primary data (which was not the case here).

### Search strategy

The systematic search was performed in PubMed, Cochrane Library, LILACS, and ScienceDirect to identify studies published up to March 2025. The search strategy combined controlled vocabulary and free-text terms related to (1) the exposures of interest (vitamin B12, vitamin D, folate/folic acid), (2) pregnancy, and (3) neurodevelopmental outcomes in children. The following Boolean structure was applied and adapted to each database:

(“Vitamin B12 deficiency” OR “Vitamin B12” OR “cobalamin” OR “Vitamin D deficiency” OR “Vitamin D” OR “25(OH) D” OR “folate” OR “folic acid” OR “folic acid supplementation”)

AND (“Pregnancy” OR “pregnant women” OR “gestation”)

AND (“Neurodevelopment” OR “child neurodevelopment” OR “cognitive development” OR “language development” OR “motor development” OR “behavior” OR “mental health”)

Filters were applied for language (English, Portuguese, Spanish) and publication period (2015–2025) in line with the review scope. Article types eligible for inclusion were original observational or interventional studies; systematic reviews and meta-analyses were retrieved for background screening and reference harvesting.

**Figure 1:**
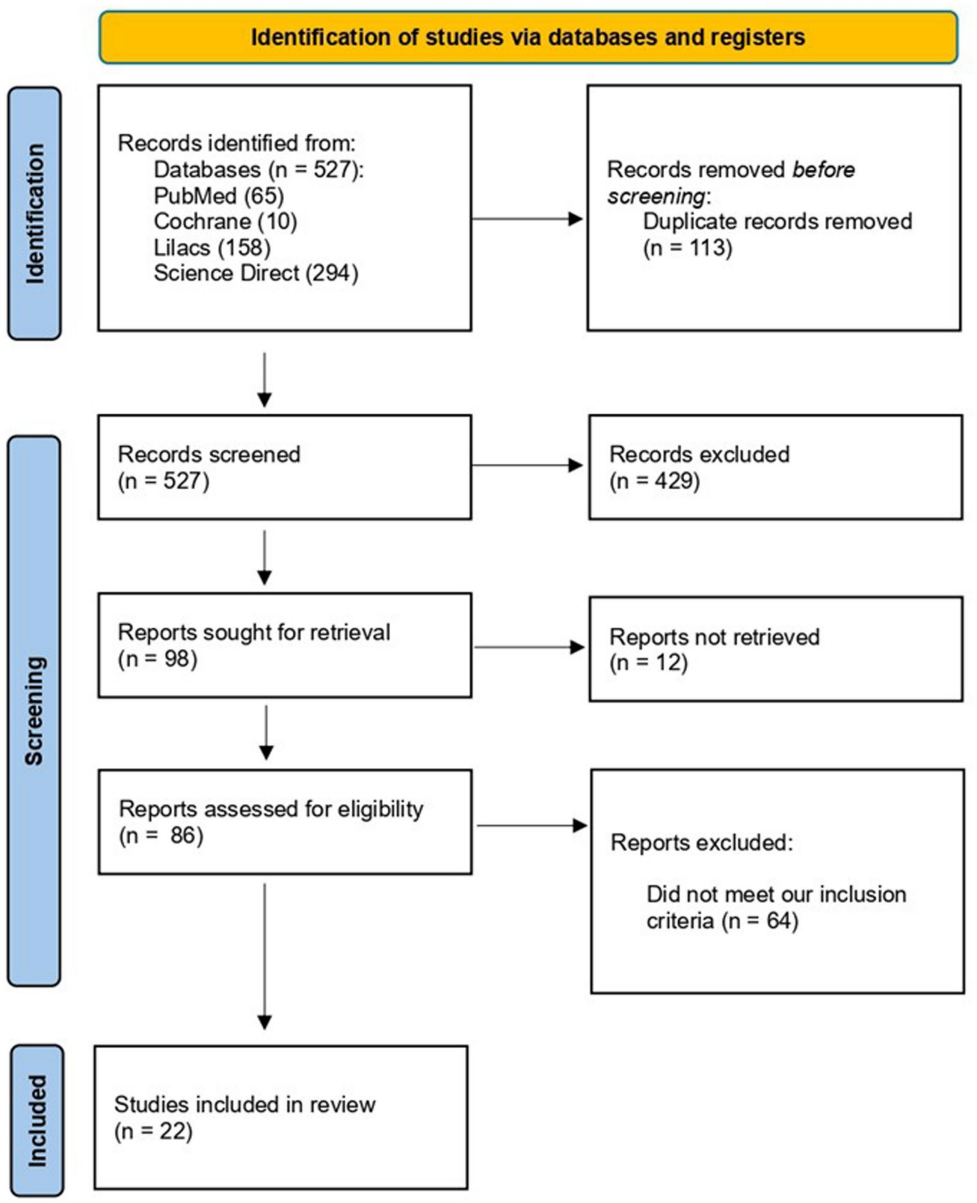
PRISMA flow diagram of study selection for maternal micronutrient status and offspring neurodevelopment studies.

The database search yielded 527 records (PubMed: 65; Cochrane Library: 10; LILACS: 158; ScienceDirect: 294). After removing duplicates (*n* = 113), the remaining records were screened by title and abstract, and 429 were excluded. Ninety-eight reports were sought for retrieval; 12 could not be retrieved. Consequently, 86 full-text articles were assessed for eligibility, and 64 were excluded for not meeting the inclusion criteria. Finally, 22 studies were included in the qualitative synthesis. The study selection process is summarized in the PRISMA flow diagram (Figure 1).

### Data selection and extraction

#### Study selection

Following the initial database search, all retrieved records were exported to a reference management software and duplicates were removed. Two independent reviewers screened titles and abstracts to identify potentially eligible studies based on the predefined inclusion and exclusion criteria. Studies that clearly did not meet the eligibility criteria were excluded at this stage.

Full-text versions of all potentially relevant articles were subsequently retrieved and assessed independently by the same reviewers. Eligibility was determined according to predefined criteria regarding population characteristics, exposure definition (maternal vitamin B12, vitamin D, or folate status or supplementation), outcome measures (neurodevelopmental outcomes), and study design. Discrepancies between reviewers were resolved through discussion, and when consensus could not be reached, a third senior reviewer was consulted.

#### Data extraction

Data extraction was performed independently by two reviewers using a standardized and piloted data extraction form. The following information was systematically collected from each included study:

- Study characteristics: author(s), year of publication, country, study design, and sample size.
- Participant characteristics: maternal age, gestational age at exposure assessment, and population characteristics.
- Exposure assessment: type of micronutrient (vitamin B12, vitamin D, folic acid), biological matrix used for assessment (e.g., serum, plasma), dosage or concentration, and timing of exposure during pregnancy.
- Outcome measures: type of neurodevelopmental assessment (cognitive, language, motor, behavioral), age at outcome assessment, and assessment tools used.
- Key findings: direction and magnitude of associations between micronutrient status and neurodevelopmental outcomes.
- Adjustment variables: confounders included in multivariable analyses (e.g., socioeconomic status, maternal education, gestational age, birth weight).

When information was missing or unclear, the original publications were reviewed in detail to ensure accurate data extraction. Any disagreements between reviewers were resolved through discussion or, when necessary, adjudicated by a third reviewer.

All studies included in the qualitative synthesis are summarized and referenced in Table 1 (Dhamayanti et al., 2019; Darling et al., 2017; Wang et al., 2023; Zhu et al., 2015; Supriadi et al., 2022; McNulty et al., 2019; Chatzi et al., 2012; Voltas et al., 2020; Yan et al., 2019; Srinivasan et al., 2017; Wang et al., 2018; Aagaard et al., 2024; Huang et al., 2020; Melough et al., 2021; Whitehouse et al., 2012; Cruz-Rodríguez et al., 2023; Suzuki et al., 2022; Lai et al., 2019; Nishigori et al., 2023; Ars et al., 2016; Chi et al., 2018; Rodgers et al., 2023).

**Table 1:** Summary of neurodevelopmental domains, assessment tools, and overall patterns of findings across studies evaluating maternal vitamin D, vitamin B12, and folate status during pregnancy.

Micronutrient	Number of studies	Main exposure assessment	Main neurodevelopmental tools	Most affected domains	Overall patterns of findings
Vitamin D	11	Maternal serum or cord blood 25(OH)D concentrations; supplementation trials	ASQ-3, Bayley Scales of Infant Development, DDST-II, Gesell Developmental Schedules, IQ assessments	Motor, psychomotor, and selected social-behavioral domains; cognitive findings less consistent	Predominantly domain-specific and inconsistent associations; stronger evidence for early motor and psychomotor outcomes
Vitamin B12	4	Maternal serum vitamin B12 concentrations; supplementation trials	Bayley Scales, language and cognitive developmental assessments	Language, attention, executive functioning	Generally modest positive associations with cognitive and language-related outcomes; limited interventional evidence
Folate / Folic acid	7	Dietary intake, supplementation records, serum and RBC folate biomarkers	Language scales, cognitive assessments, executive function measures	Cognition, language, behavioral, and executive functioning outcomes	Most consistent associations across populations and study designs, particularly during the periconceptional period

ASQ-3, Ages and Stages Questionnaire; DDST-II, Denver Developmental Screening Test II; RBC, red blood cell.

### Quality assessment and risk of Bias

#### Assessment tools

Methodological quality and risk of bias were evaluated using validated tools appropriate to study design:

- Randomized controlled trials (RCTs) were assessed using the Jadad scale, which evaluates randomization procedures, blinding, and reporting of withdrawals or dropouts. Studies scoring ≥3 were considered of high quality.
- Observational studies (cohort and case–control) were assessed using the Newcastle–Ottawa Scale (NOS), which evaluates selection of participants, comparability of study groups, and ascertainment of outcomes. Studies achieving a score of ≥7 were classified as high quality (Jadad et al., 1996; Wells et al., 2014).

#### Quality classification and interpretation

Based on these assessments, studies were categorized as high, moderate, or low quality. Results from studies rated as high quality were given greater interpretative weight in the synthesis, while findings from studies with methodological limitations were interpreted cautiously. Attention was given to potential sources of bias, including residual confounding, misclassification of exposure, and variability in outcome assessment tools.

#### Assessment of Bias and methodological limitations

Potential sources of bias were systematically evaluated, including selection bias, information bias, and confounding. Specific attention was given to (i) variability in biomarker assessment methods and cut-off values for vitamin deficiencies; (ii) differences in timing of exposure assessment during pregnancy; (iii) inconsistencies in neurodevelopmental assessment tools and outcome definitions; and (iv) residual confounding related to socioeconomic status, maternal education, diet quality, and co-existing micronutrient deficiencies.

The potential influence of publication bias was considered qualitatively through comparison of findings across study designs and sample sizes, although formal statistical assessment (e.g., funnel plots) was not feasible due to heterogeneity.

## Results

### Study selection and characteristics

The study selection process is illustrated in Figure 1. A total of 527 records were identified through database searches. After duplicate removal (*n* = 113) and title/abstract screening (429 excluded), 98 reports were sought for retrieval, of which 12 could not be retrieved. Eighty-six full-text articles were assessed for eligibility, and 64 were excluded. Overall, 22 primary studies were included in the qualitative synthesis, comprising 18 observational studies (81.8%) and 4 randomized controlled trials (18.2%) (Supplementary Figure S1).

Studies were conducted across diverse geographical regions, including Asia, Europe, and North America, and predominantly consisted of prospective cohort studies evaluating maternal or cord blood micronutrient status during pregnancy. Sample sizes varied substantially, ranging from small cohorts to large population-based studies involving several thousand mother–child pairs.

Neurodevelopmental outcomes were evaluated using validated instruments measuring cognitive, motor, language, and behavioral domains. Across studies, outcome assessment tools included Bayley Scales of Infant Development, ASQ-3, DDST-II, Gesell Developmental Schedules, standardized IQ measures, and parent- or clinician-reported behavioral assessments. The age at assessment ranged from infancy to late childhood, contributing to variability in outcome measurement but allowing evaluation of both early and later neurodevelopmental effects.

Vitamin D was the most frequently investigated micronutrient among the included studies, followed by vitamin B12 and folate-related exposures (Supplementary Figure S2).

The relatively small number of randomized controlled trials limited definitive causal interpretation of the available evidence.

To facilitate comparison across micronutrients and neurodevelopmental domains, Table 1 summarizes the principal methodological characteristics, neurodevelopmental assessment tools, and overall patterns of findings identified across the included studies.

Although formal quantitative meta-analysis was not feasible due to substantial methodological heterogeneity, semiquantitative consistency patterns across neurodevelopmental domains could be identified. Overall, folate-related studies demonstrated the most consistent associations across populations and developmental domains, whereas vitamin D findings were more heterogeneous and frequently domain-specific. Evidence regarding vitamin B12 remained comparatively limited, although several studies suggested modest associations with language and cognitive outcomes.

### Vitamin D and neurodevelopmental outcomes

Among the included vitamin D studies, most reported at least one association between maternal or cord blood vitamin D status and specific neurodevelopmental domains, although findings were frequently domain-specific and inconsistent across follow-up periods. The most consistent associations involved motor and psychomotor outcomes during infancy and early childhood, whereas associations with later cognitive performance and IQ were less consistent.

Several prospective cohort studies identified associations involving motor, psychomotor, or social outcomes during early childhood, although persistence across later developmental stages was less consistently observed. Associations with later cognitive performance, including IQ and reading outcomes, were often attenuated after adjustment for confounding variables.

Considerable methodological diversity was observed across studies in terms of exposure definition, timing of biomarker assessment, neurodevelopmental instruments, and follow-up duration. Vitamin D exposure was assessed using maternal serum concentrations, cord blood biomarkers, or supplementation protocols, with substantial variation in deficiency thresholds and categorization methods. Neurodevelopmental outcomes were evaluated using multiple instruments, including ASQ-3, Bayley Scales of Infant Development, DDST-II, Gesell Developmental Schedules, and standardized IQ measures. This methodological diversity likely contributed to variability in reported associations and limited direct comparison across neurodevelopmental outcomes.

Some studies suggested more complex biological relationships beyond a simple deficiency model, including nonlinear associations and potential modulatory effects involving inflammatory and metabolic pathways.

Randomized controlled trials evaluating maternal vitamin D supplementation produced more variable findings than observational studies. While some trials demonstrated modest improvements in motor or cognitive outcomes, others reported null associations. Differences in baseline nutritional status, supplementation dose, gestational timing, and duration of intervention likely contributed to these inconsistencies.

Overall, the available evidence suggests that vitamin D may influence early neurodevelopment, particularly in motor-related domains, although findings remain inconsistent and causality cannot be firmly established due to methodological variability and the predominance of observational evidence.

### Vitamin B12 and neurodevelopmental outcomes

Evidence regarding maternal vitamin B12 status and offspring neurodevelopment was more limited compared with vitamin D and folate. Nevertheless, most vitamin B12 studies identified associations involving language, attention, or cognitive domains, although effect sizes were generally modest and methodological heterogeneity remained substantial. Both observational studies and randomized controlled trials were identified, including populations with relatively high prevalence of maternal B12 deficiency.

Across studies, lower maternal vitamin B12 concentrations were generally associated with less favorable domain-specific neurodevelopmental performance, particularly involving language, attention, memory, and executive functioning outcomes, although effect sizes were frequently modest. Some randomized controlled trials evaluating maternal vitamin B12 supplementation demonstrated modest improvements in cognitive and language-related outcomes, supporting a potential biological role for adequate B12 availability during fetal neurodevelopment.

However, findings were not entirely consistent across studies, and effect sizes were often modest. Considerable methodological variability was observed in exposure assessment, supplementation protocols, developmental instruments, and follow-up periods. In addition, several studies relied on single biomarker measurements obtained during pregnancy, limiting assessment of long-term maternal nutritional status and increasing susceptibility to residual confounding.

Some studies also evaluated vitamin B12 in combination with folate-related pathways, reflecting the close biological interaction between these micronutrients in one-carbon metabolism and methylation processes. Nevertheless, evidence specifically investigating combined micronutrient effects remained limited.

Overall, the available evidence suggests that maternal vitamin B12 status may contribute to offspring neurodevelopment, particularly in cognitive and language-related domains, although the relatively small number of studies and methodological variability limits definitive conclusions regarding causality and long-term effects.

### Folate and neurodevelopmental outcomes

Among the included micronutrients, folate-related studies demonstrated the most consistent associations across cognitive, language, behavioral, and executive functioning domains. Evidence from both prospective cohort studies and randomized controlled trials suggested that adequate maternal folate status, particularly during the periconceptional and early gestational periods, was associated with more favorable neurodevelopmental outcomes in offspring.

Several studies reported that children born to mothers with sufficient folate intake or supplementation presented more favorable neurodevelopmental trajectories during infancy and childhood. These associations were observed across multiple developmental domains, including language acquisition, attention, social functioning, and cognitive performance. In contrast to the more inconsistent findings observed for vitamin D, folate-related associations appeared more consistent across populations and study designs.

The timing of exposure emerged as a particularly important factor. Associations were generally stronger when folic acid supplementation occurred before conception or during early pregnancy, supporting the importance of folate availability during critical periods of neural tube formation, neuronal proliferation, and early brain development. Some studies also suggested that prolonged supplementation beyond the first trimester may provide additional neurodevelopmental benefits, although findings in this area remained less consistent.

Despite the overall consistency of findings, methodological diversity remained substantial. Studies differed in folate assessment methods, including dietary intake estimation, supplementation records, serum biomarkers, and red blood cell folate concentrations. Neurodevelopmental outcomes were also evaluated using multiple assessment instruments and follow-up periods, limiting direct comparison across studies.

In addition, some observational studies evaluating folate-related pathways also incorporated vitamin B12 status, reflecting the biological interdependence of these micronutrients within one-carbon metabolism and methylation processes. However, relatively few studies formally investigated combined micronutrient interactions or genetic polymorphisms potentially influencing folate metabolism.

Overall, the available evidence supports a potentially important role for adequate maternal folate status in early neurodevelopment, particularly during critical prenatal developmental windows. Nevertheless, residual confounding, variability in exposure assessment, and the predominance of observational evidence continue to limit definitive causal interpretation.

**Figure 2:**
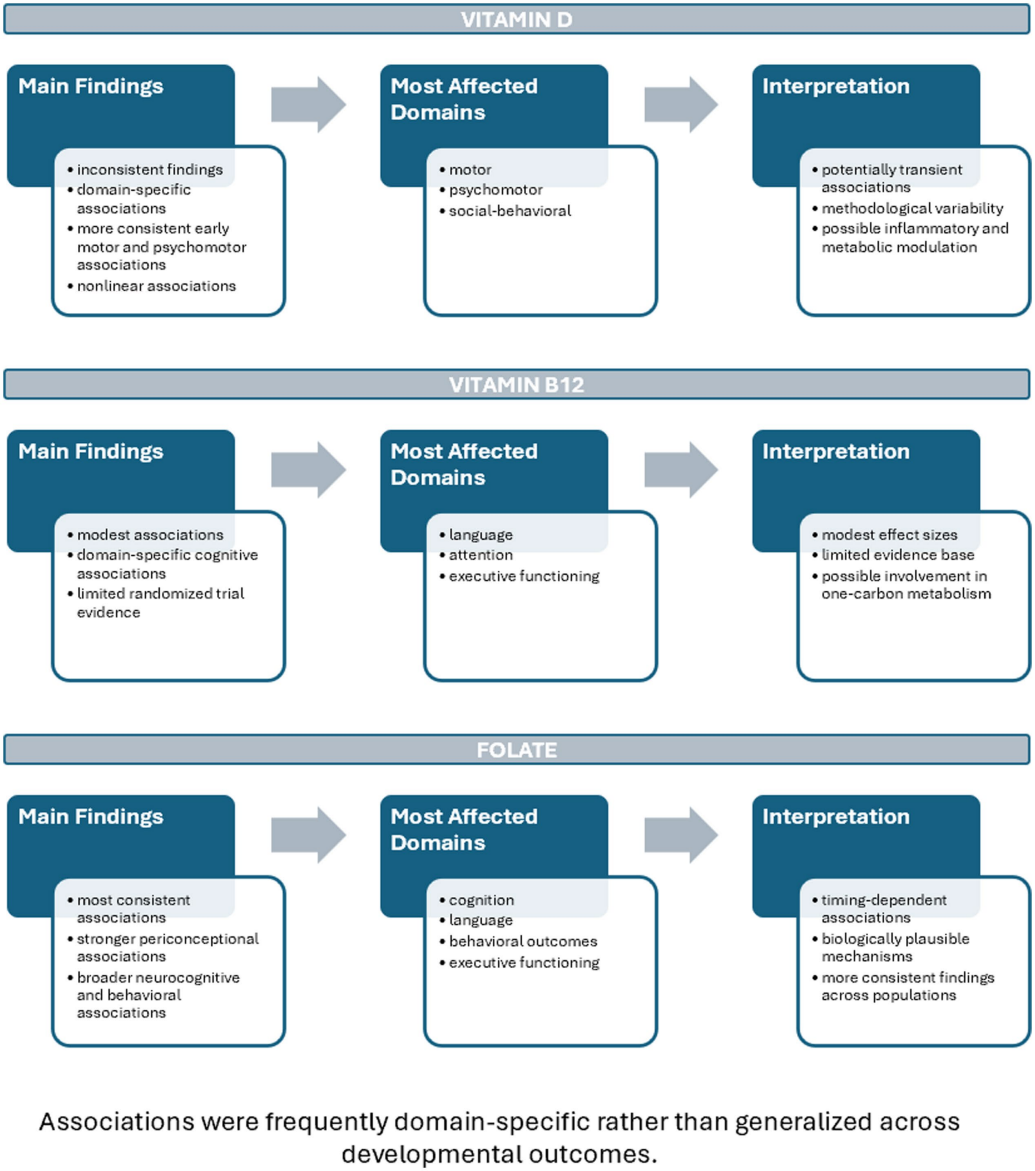
Summary of neurodevelopmental patterns associated with maternal vitamin D, vitamin B_12_, and folate status.

A conceptual summary of the main neurodevelopmental patterns associated with maternal vitamin D, vitamin B12, and folate status across the included studies is presented in Figure 2.

## Discussion

This systematic review synthesized current evidence regarding the relationship between maternal vitamin B12, vitamin D, and folate status during pregnancy and neurodevelopmental outcomes in offspring. Overall, the available evidence suggests that adequate maternal availability of these micronutrients during critical periods of gestation may contribute to more favorable neurodevelopmental trajectories, particularly in cognitive, language, and selected motor domains. However, associations were frequently domain-specific rather than generalized across all developmental outcomes, and substantial methodological variability was observed across studies. Differences in exposure assessment, timing of evaluation, neurodevelopmental instruments, and population characteristics likely contributed to inconsistencies in the available evidence. Nevertheless, the overall findings remain biologically plausible and clinically relevant, particularly in the context of contemporary dietary patterns characterized by high consumption of ultra-processed foods and reduced micronutrient density (Prado and Dewey, 2014; Cusick and Georgieff, 2016; Nyaradi et al., 2013; Bailey et al., 2015; Black et al., 2013).

Vitamin D demonstrated a more inconsistent relationship with neurodevelopmental outcomes compared with folate. Several observational studies identified associations between low maternal or cord blood 25-hydroxyvitamin D concentrations and impairments in motor, psychomotor, social, and behavioral domains during early childhood. However, these associations were frequently domain-specific and were not consistently maintained at later developmental assessments, suggesting potentially transient associations rather than stable long-term neurocognitive differences. Randomized controlled trials yielded mixed findings, likely reflecting variability in baseline nutritional status, supplementation protocols, timing of exposure, and neurodevelopmental assessment methods. Some studies also suggested more complex biological mechanisms, including nonlinear associations and potential inflammatory or metabolic modulation. Collectively, the available evidence suggests a biologically plausible but methodologically inconsistent relationship between vitamin D status and neurodevelopment (McCann and Ames, 2008; McGrath et al., 2010; Morales et al., 2015; Strøm et al., 2014; Darling et al., 2017; Voltas et al., 2020; Wang et al., 2018; Whitehouse et al., 2012). Compared with folate-related findings, vitamin D associations appeared substantially more heterogeneous and more susceptible to methodological variability across studies.

Maternal vitamin B12 status emerged as a potentially important contributor to offspring neurodevelopment, although the available evidence base remains relatively limited compared with vitamin D and folate. Across observational studies and randomized trials, lower maternal B12 concentrations were associated with less favorable cognitive, language, attention, and executive functioning outcomes in children. These findings are biologically coherent given the established role of vitamin B12 in one-carbon metabolism, DNA synthesis, myelination, and neurotransmitter production. Evidence from randomized controlled trials provides partial support for a potential causal contribution, particularly in populations with high prevalence of maternal B12 deficiency. However, modest effect sizes, differences in exposure assessment, and the limited number of interventional studies continue to restrict definitive causal interpretation (Black, 2008; Dror and Allen, 2008; Aparicio et al., 2020).

Compared with vitamin D and vitamin B12, folate-related associations appeared more consistent across populations and study designs. Adequate maternal folate status, particularly during the periconceptional and early gestational periods, was associated with more favorable cognitive, language, behavioral, and executive functioning outcomes in offspring. These findings are biologically plausible given the central role of folate in DNA synthesis, methylation pathways, neuronal proliferation, and early neurogenesis. The timing of exposure appeared especially important, with stronger associations observed when folic acid supplementation occurred before conception or during early pregnancy. Nevertheless, differences in exposure assessment methods, follow-up periods, and neurodevelopmental instruments continue to limit direct comparison across studies and restrict definitive causal interpretation (Czeizel et al., 2011; Julvez et al., 2009; Roth et al., 2011; Surén et al., 2013; McNulty et al., 2019; Chatzi et al., 2012; Yan et al., 2019; Huang et al., 2020; Suzuki et al., 2022; Nishigori et al., 2023).

The timing of micronutrient exposure likely represents a critical determinant of neurodevelopmental vulnerability, particularly during periods of rapid neuronal proliferation, synaptogenesis, and epigenetic programming.

**Figure 3:**
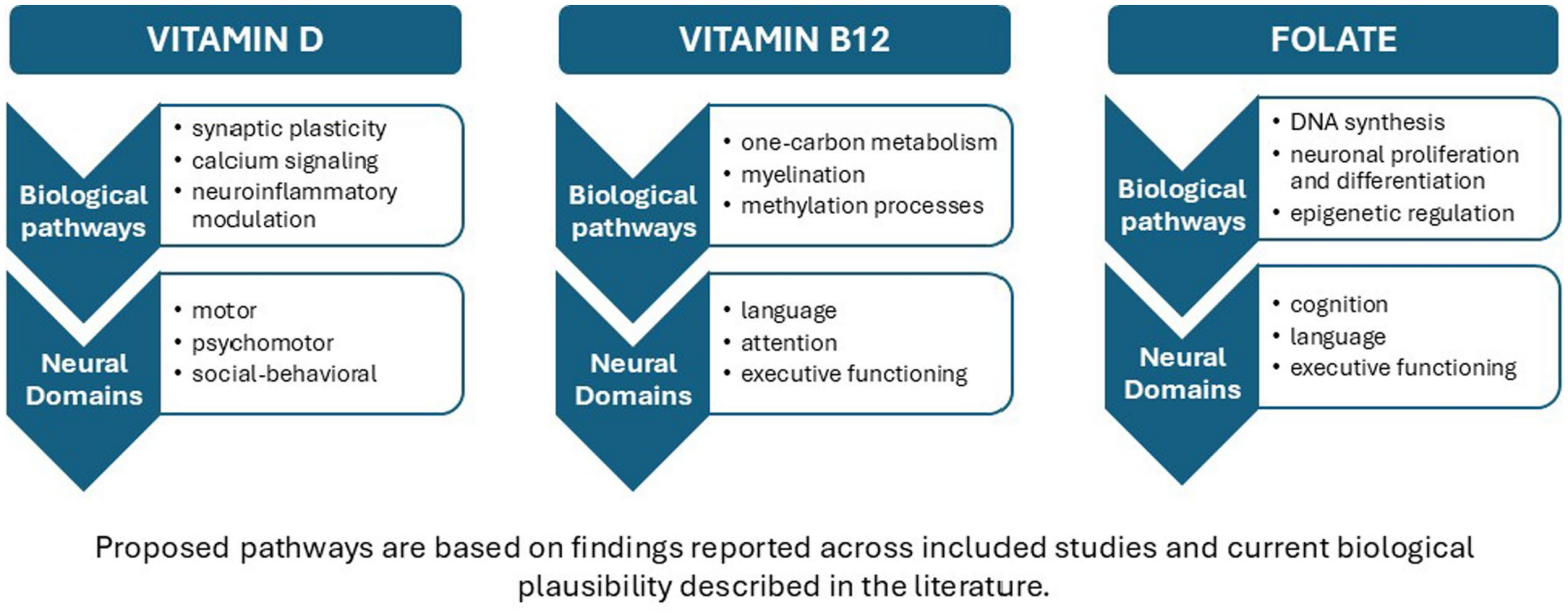
Biological pathways linking maternal micronutrient status to offspring neurodevelopment.

Although discussed separately, these micronutrients likely interact within interconnected biological pathways involved in fetal brain development. Folate and vitamin B12 participate directly in one-carbon metabolism and methylation processes, while vitamin D may exert modulatory effects through neuroimmune and inflammatory pathways. Consequently, isolated micronutrient analyses may not fully capture the complexity of prenatal nutritional influences on neurodevelopment. Proposed biological pathways potentially linking maternal micronutrient status to offspring neurodevelopment are summarized in Figure 3.

The results of this review are consistent with large cohort studies such as the ECLIPSES and Generation R studies, which reported associations between maternal micronutrient status and later neurodevelopmental outcomes. However, the strength and consistency of these associations varied across studies, reflecting important differences in study design, exposure assessment, outcome measurement, and population characteristics (Aparicio et al., 2020; Darling et al., 2017; Whitehouse et al., 2012; Ars et al., 2016).

Importantly, the reviewed evidence highlights that micronutrient deficiencies rarely occur in isolation. In many populations, deficiencies in vitamin B12, vitamin D, and folate coexist, particularly in settings characterized by high consumption of ultra-processed foods. Such dietary patterns are often energy-dense but micronutrient-poor, contributing to a phenomenon sometimes referred to as “hidden hunger.” This context may partially explain the variability in findings across studies and underscores the need to consider overall dietary quality rather than isolated nutrient effects (Bailey et al., 2015; Black et al., 2013).

Interpretation of the available evidence is further complicated by the predominance of observational studies, which remain particularly susceptible to residual confounding and variability in exposure assessment. These include variability in the timing of exposure assessment, differences in biomarker thresholds used to define deficiency, and diversity in neurodevelopmental assessment tools. The use of different developmental instruments across studies may have contributed not only to methodological variability but also to differences in sensitivity for detecting domain-specific neurodevelopmental effects. Additionally, many observational studies faced challenges related to residual confounding, particularly socioeconomic status, maternal education, and overall dietary quality.

The timing of exposure appears especially critical. Because maternal exposure preceded outcome assessment in most included studies, reverse causality is less likely to explain the observed associations. Evidence suggests that deficiencies occurring during early gestation may have more profound and lasting effects on neurodevelopment than deficiencies occurring later in pregnancy. Furthermore, genetic variability—such as polymorphisms affecting folate metabolism—may modify individual susceptibility and partially explain inconsistent findings across populations.

From a clinical perspective, the findings reinforce the importance of comprehensive nutritional assessment during pregnancy, beyond routine folate supplementation alone. Monitoring maternal vitamin B12 and vitamin D status may help identify pregnancies potentially at increased risk for adverse neurodevelopmental outcomes.

From a public health standpoint, the results highlight the need to reassess current nutritional policies in light of changing dietary patterns. The widespread consumption of ultra-processed foods, often poor in essential micronutrients, may undermine existing supplementation strategies. Strengthening nutritional education, improving access to nutrient-dense foods, and considering targeted supplementation programs could represent effective strategies to mitigate these risks, particularly in vulnerable populations (Bailey et al., 2015; Black et al., 2013).

The substantial variability in exposure definitions, biomarker thresholds, timing of assessment, and neurodevelopmental outcome measures precluded formal quantitative meta-analysis and limited direct comparison across studies, supporting the use of a qualitative synthesis approach.

Despite these limitations, this review presents several strengths, including a comprehensive search strategy, inclusion of both observational and interventional evidence, and a structured critical synthesis of neurodevelopmental domains across multiple micronutrients. Nevertheless, important limitations should be acknowledged. Most included studies originated from high-income settings, potentially limiting generalizability to low- and middle-income populations where micronutrient deficiencies may be more prevalent and dietary patterns substantially different. Additionally, the predominance of observational evidence restricts definitive causal interpretation.

Future research should prioritize well-designed longitudinal studies and randomized controlled trials using standardized exposure assessments and harmonized neurodevelopmental outcome measures. Greater emphasis should also be placed on evaluating combined micronutrient interactions, critical developmental windows, and potential gene–nutrient interactions that may influence susceptibility to adverse neurodevelopmental outcomes.

## Conclusion

In conclusion, the available evidence suggests that maternal vitamin B12, vitamin D, and folate status during pregnancy may influence offspring neurodevelopmental outcomes through complex and potentially interconnected biological pathways. Folate-related findings demonstrated the greatest consistency across populations and developmental domains, whereas vitamin D associations appeared comparatively more heterogeneous and vitamin B12 evidence remained more limited. Although findings were frequently domain-specific and methodologically heterogeneous, the overall evidence reinforces the potential importance of adequate maternal micronutrient status during critical periods of fetal brain development. Beyond the established role of folate in neural tube defect prevention, emerging evidence suggests that interconnected micronutrient pathways may contribute to broader neurodevelopmental trajectories. These findings reinforce the importance of broader nutritional surveillance and preventive strategies during pregnancy, while also underscoring the need for further high-quality longitudinal and interventional studies.

## Data Availability

The original contributions presented in the study are included in the article/Supplementary material, further inquiries can be directed to the corresponding author.
