# Supplementary figures and images for: Maternal vitamin B12, vitamin D, and folic acid status during pregnancy and child neurodevelopment: a systematic review
Source: Front Neurosci. 2026 Jun 10;20:1825297. doi: 10.3389/fnins.2026.1825297 (PMC13290869; doi:10.3389/fnins.2026.1825297)

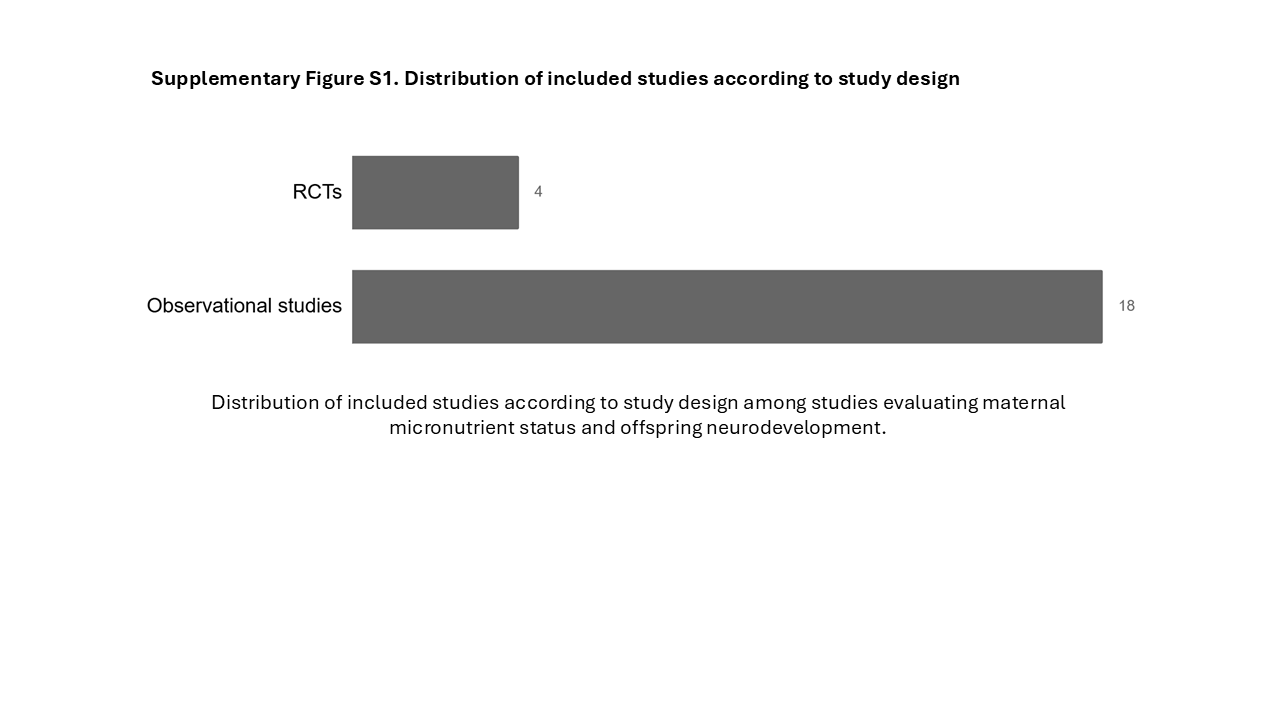

Supplement: Supplementary file 1 [file Image_1.TIF]

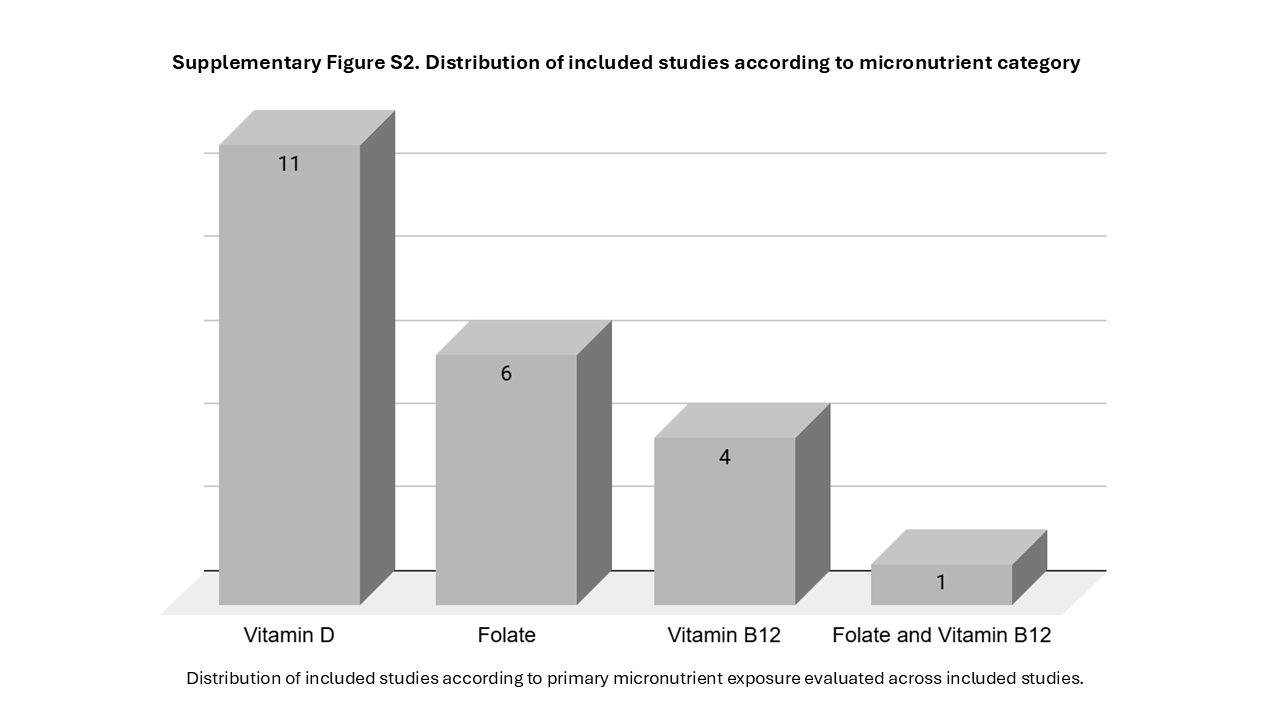

Supplement: Supplementary file 2 [file Image_2.TIF]
